# A Rare Case of Metastatic Small Cell Carcinoma of Lung in a Follow-Up Patient of Chronic Myeloid Leukemia on Imatinib Treatment

**DOI:** 10.7759/cureus.35436

**Published:** 2023-02-24

**Authors:** Anurag Singh, Nida Shabbir, Tanya Tripathi, Rashmi Kushwaha, Shailendra P Verma

**Affiliations:** 1 Pathology, King George's Medical University, Lucknow, IND; 2 Clinical Hematology, King George's Medical University, Lucknow, IND

**Keywords:** tyrosine kinase receptor inhibitors, small-cell lung carcinoma, imatinib, tyrosine kinase inhibitor, chronic myeloid leukaemia

## Abstract

Imatinib has an excellent long-term survival rate and significantly ameliorates the treatment of chronic myeloid leukaemia during the past few decades. There is now a concern that first-generation tyrosine kinase inhibitors can cause secondary neoplasms. Here, we describe a case of a 49-year-old non-smoker male who was diagnosed with chronic myeloid leukaemia and treated with imatinib. After 15 years of treatment, an incidental right cervical lymphadenopathy was noted. The fine needle aspiration cytology from the lymph node revealed the small round cell morphology. In order to identify the primary lesion, computerised tomography of the thorax and abdomen was advised, which revealed a diagnosis of small cell carcinoma lung. In the index case report, we will discuss the potential side effects of first-generation tyrosine kinase inhibitors on a long-term basis along with treatment protocols for metastatic small cell carcinoma lung in a disease-free follow-up case of chronic myeloid leukaemia.

## Introduction

Tyrosine kinase inhibitors (TKIs) that target the BCR-ABL protein have considerably reduced the progression rate of chronic myeloid leukaemia (CML) [1]. There is excellent long-term survival with imatinib, with a survival rate of 90% and 88% after five and eight years, respectively [2]. The risk of secondary malignancies rises with exposure to chemotherapy and immunosuppression [3,4]. The interactivity of imatinib with DNA repair mechanisms has been demonstrated in preclinical studies [5,6]. Imatinib also suppresses T-lymphocyte activity and prevents the development of peripheral blood progenitor cells into dendritic cells [7]. The acquired translocation t(9;22) at diagnosis and further chromosomal mutations as a result of clonal evolution raise the possibility of genetic instability in CML. Progenitors may therefore be able to establish themselves as distinct cell types with increased malignancy either before or after CML, leading to solid tumours or haematological malignancies [8]. In this case report, small-cell lung cancer (SCLC) developed in a known CML patient on imatinib treatment, which is extremely unusual.

## Case presentation

A 49-year-old man, a follow-up case of CML diagnosed in the year 2007, was on regular imatinib (400 mg/day) treatment. After 15 years of disease-free time period, he was found to have incidental right cervical lymphadenopathy on physical examination during a follow-up visit on April 2022. The lymph node was of size 1x0.8x0.8 cm and firm, mobile, and non-tender. Abdominal palpation and ultrasonography both did not reveal any organomegaly. There was no history of substance abuse in form of tobacco chewing and smoking. There was no history of cough, haemoptysis, or breathlessness.

**Figure 1:**
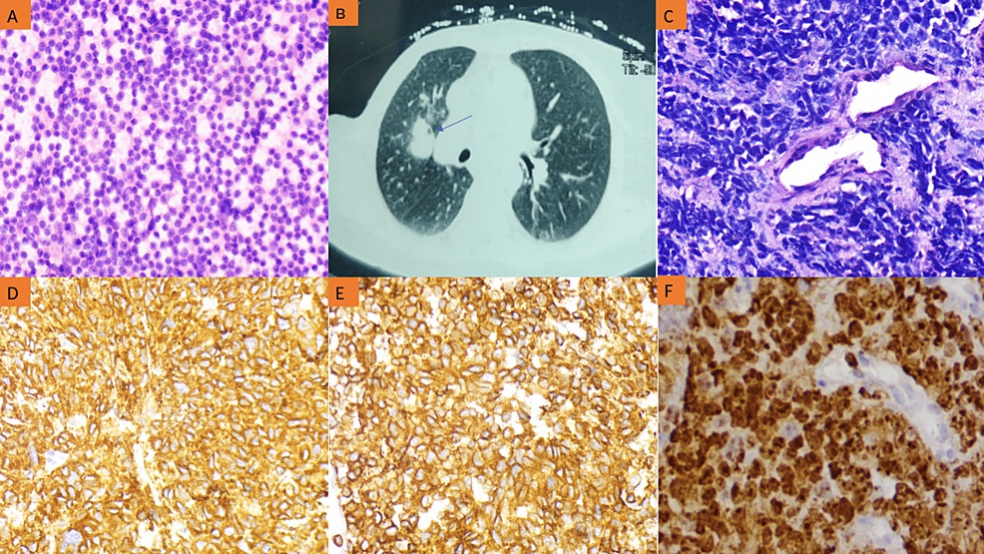
Photomicrograph of right cervical lymph node cytology smear, computed tomography of the thorax, lung biopsy, and immunohistochemistry (A) Fine needle aspiration cytology smear from a cervical lymph node showing singly dispersed atypical cells having enlarged nuclei and ill-defined eosinophilic cytoplasm, (x200), H&E stain; (B) Contrast-enhanced computed tomography of the thorax displaying heterogeneous enhancing soft tissue mass in the upper lobe of the right lung; (C) H&E stained section of biopsy from lung mass revealed solid growth of round to oval atypical cells displaying hyperchromatic nuclei, inconspicuous nucleoli and a scant amount of cytoplasm (x400); (D) Immunohistochemistry showing a positive expression of synaptophysin; (E) Chromogranin; (F) Ki-67 proliferation index of ~95% among tumour cells (x400) H&E: hematoxylin and eosin

Fine needle aspiration cytology (FNAC) was performed from the right cervical lymph node, which showed cellular smears composed of atypical cells having round to oval hyperchromatic nuclei and a scant amount of cytoplasm on a haemorrhagic background (Figure 1A). On the basis of cytology findings, the possibility of primary neuroendocrine neoplasm or metastasis from small cell carcinoma was kept.

In order to identify the primary lesion, contrast-enhanced computerised tomography (CECT) of the thorax and abdomen was advised, which revealed heterogeneous enhancing soft tissue mass in the upper lobe of the right lung which measured 35x25 mm. There was no cavitation or calcification seen. No mediastinal lymphadenopathy was noted (Figure 1B). For a definitive diagnosis, a bronchoscopy-guided biopsy from lung mass and immunohistochemistry were advised.

Histopathological examination of the Hematoxylin and eosin (H&E)-stained section of the biopsy from the lung mass showed small-sized atypical cells with coarse chromatin, inconspicuous nucleoli, and a scant amount of cytoplasm. A few tumour cells showed nuclear moulding and crush artefacts (Figure 1C). Immunohistochemistry revealed strong immunoreactivity towards synaptophysin and chromogranin (Figures 1D, 1E). Ki67 proliferation index was high (~95%) (Figure 1F). The tumour cells also showed expression of CD56 and TTF-1 with weak focal positivity for cytokeratin (CK) 7. The tumour cells were negative for p63, p40, CK20, and napsin. On the basis of histomorphology and immunohistochemistry, a definitive diagnosis of SCLC was rendered.

The patient was planned for the treatment of metastatic SCLC, and for that, he received six cycles of combined platin-based chemotherapy and radiotherapy. The cervical lymphadenopathy disappeared and the patient progressed favourably and continued on oral dasatinib. The patient was in regular follow-up for six months and currently doing well.

## Discussion

Imatinib is a first-generation TKI that inhibits Bcr-Abl tyrosine kinase (TK) and is used in treating CML. It is important for clinicians to be aware of secondary cancers in their daily practice because they have been reported in a number of cases utilising TKIs [9]. A study from the National Cancer Institute analysed 8005 patients with CML who were diagnosed between 1973 and 2000. They found an increase of 16% and a cumulative incidence of new cancer of 4.8% in CML cases [10]. Few studies have shown that first-generation TKIs are a possible risk factor linked to both solid tumours and secondary haematological malignancies (Table 1).

**Table 1:** Studies showing second malignancies in follow-up cases of CML that were treated with first-generation TKIs CML: chronic myeloid leukaemia; TKI: tyrosine kinase inhibitor

S.N.	Total number of CML cases	Treatment	Follow-up (Month)	Number of second malignancy cases	Types of second malignancies	Year	Author
1.	2753	Imatinib	24	125	Urogenital tract carcinoma-39; Melanoma of skin-3; Other skin cancer-18; Prostate adenocarcinoma-17; Colon/rectum adenocarcinoma-11; Stomach adenocarcinoma-10; Myeloid leukemia-9; Non-Hodgkin’s lymphoma-5; Lymphoid leukemia-5; Lung and bronchus carcinoma-6; Buccal cavity squamous cell carcinoma-2	2010	Rebora et al. [11]
2.	1525	Imatinib	67.5	67	Prostate adenocarcinoma-9; Non-Hodgkin’s lymphoma -7; Colorectal cancer-6; Lung carcinoma-6; Malignant melanoma-5; Skin tumour-5; Breast cancer-5; Pancreas adenocarcinoma-4; Renal cell carcinoma-4; Chronic lymphocytic leukemia- 3; Head and neck carcinoma-2; Biliary adenocarcinoma-2; Sarcoma-2; Oesophagus carcinoma-1; Stomach adenocarcinoma-1; Liver carcinoma-1; Vulval carcinoma-1; Uterine carcinoma-1; Brain neoplasm-1; Cancer of unknown origin-1	2016	Miranda et al. [2]
3.	339	Imatinib	40 month	14	Stomach adenocarcinoma-3; Colon adenocarcinoma-2; Prostate adenocarcinoma-1; Breast cancer-1; Lung carcinoma-1; Liver carcinoma-1; Ovary adenocarcinoma-1; Larynx carcinoma-1; Oesophagus carcinoma-1; Tongue squamous cell carcinoma-1; Non-Hodgkin’s lymphoma-1	2018	Nakazato et al. [12]
4.	189	Imatinib	5-96	6	Prostate adenocarcinoma-3; Urinary bladder carcinoma-1; Colon adenocarcinoma-1; Unknown primary-1	2005	Roy et al. [13]

Secondary malignancies were found in 4.2% (64/1525) of the CML cases treated with TKI in a study by Miranda et al. Prostate, non-Hodgkin's lymphoma (NHL), colorectal, lung, malignant melanoma, non-melanoma skin tumours, and breast cancer were the most prevalent malignancies. They found lung carcinoma in 0.39% (6/1525) of CML cases that were treated with imatinib [2]. Rebora et al. found increased risks of urogenital, gastrointestinal, and skin cancers among individuals with CML diagnosed between 1970 and 1995 based on data from the Swedish national cancer registry. In their study, they observed 0.22% (6/2753) cases of lung and bronchus carcinoma treated with imatinib [11]. Most patients with second malignancies were treated with imatinib as their first-line treatment, and second-generation TKIs were not included in the treatment. As reported by Nakazato et al., patients receiving dasatinib or nilotinib treatment for CML were less likely to develop second malignancies. In their study, they noted 0.29% (1/339) cases of lung carcinoma in patients of CML with imatinib treatment [12].

It was found that imatinib contributes to secondary cancer risk. French investigators, who observed 3.2% (6/189) cases of patients treated with imatinib over a five-year period, found that they developed a second malignancy but none developed lung carcinoma [13]. In addition, the Imatinib Long-Term Side Effects (ILTE) study, a multicentre systematic review carried out by 27 centres globally, reported 3.6% (30/832) cases developed second cancers in patients of CML treated for six years with Imatinib [14]. The patient in the current report was on first-line TKI for 15 years when he developed a second malignancy in the lung.

More than 70% of patients who are diagnosed with SCLC present with metastasized disease [15]. Cancer develops from neuroendocrine precursor cells and is characterised by its rapid growth and early metastasis. Platinum and etoposide are used in conjunction as first-line therapy for metastatic SCLC [16]. However, the majority of patients relapse during the first year of treatment, which has a poor survival effect. The use of multiple agents or the inclusion of a third chemotherapy drug does not improve the results. The curation rate is still poor, even for patients who did not have metastases at diagnosis [17]. No such protocols exist for the treatment of SCLC in a disease-free follow-up case of CML on imatinib treatment.

In the present case, the patient had no symptoms of pulmonary disease despite having metastasis to the cervical lymph node. This made it challenging for the clinician to search for a primary lesion in a disease-free follow-up case of chronic myeloid leukaemia on long-term imatinib treatment.

## Conclusions

Considering the improved survival and longevity of patients with chronic myeloid leukaemia, age-appropriate cancer screening with meticulous physical examination is essential. Long-term follow-up is needed for patients receiving imatinib as front-line therapy in cases of chronic myeloid leukaemia.
